# The epidemiology of spinal fractures: A nationwide data-based study in Iran

**DOI:** 10.5339/qmj.2024.42

**Published:** 2024-09-16

**Authors:** Soroush Najdaghi, Reza Azizkhani, Neda Al-Sadat Fatemi, Mehdi Nasr Isfahani, Payman Salamati

**Affiliations:** 1 Department of Emergency Medicine, School of Medicine, Isfahan University of Medical Sciences, Isfahan, Iran; 2 Trauma Data Registration Center, Al-Zahra University Hospital, Isfahan University of Medical Sciences, Isfahan, Iran; 3 Department of Health in Disaster and Emergencies, School of Management and Medical Information Sciences, Isfahan University of Medical Sciences, Isfahan, Iran; 4 Sina Trauma and Surgery Research Center, Tehran University of Medical Sciences, Tehran, Iran *Email: m_nasr54@med.mui.ac.ir

**Keywords:** Spinal cord injury, spine, blunt trauma, Iran

## Abstract

**Background:**

Blunt trauma is a physical injury to a part of the body, mainly caused by road accidents, direct blows, attacks, sports injuries, and falls in elderly people. Spinal fractures are observed only in a small percentage of injured patients. Accordingly, the present study was conducted on collected data between 2018 and 2022 to determine the frequency of spinal fractures in blunt trauma in Iran while also considering the mechanism of injury as a secondary outcome of interest.

**Methods:**

In this retrospective study, blunt trauma patients with spinal fractures, regardless of age were included by the census sampling method. Data were obtained from the National Trauma Registry of Iran. Means and standard deviations were used for continuous variables, and the chi-square test was used to assess the relationship between the variables.

**Results:**

Among 25,986 cases of all-cause trauma patients, 1,167 cases (4.5%) of blunt trauma and spinal fracture were included in the study. Gender, the severity of injury, and the cause of trauma showed a significant difference among different age groups (p < 0.05). Significant differences were found in the injury mechanisms across various spine regions (p < 0.05). The majority of patients (68.2%) had lumbar spinal fractures. Road traffic collisions were the most common cause of spinal cord injuries, accounting for 58.3% of cases, followed by falls (36.1%). The injury severity score was higher in younger patients (under 18 years old), with a mean of 4.4 ± 3.5, and in patients with cervical injuries. The majority of injuries occurred in the lumbar area (68.2%), followed by the thoracic area. Furthermore, notable variations existed in Emergency Room (ER) stay duration, overall hospitalization, Intensive Care Unit (ICU) stay duration, and injury severity levels, all influenced by the spinal regions (*p* < 0.05). Distinctively, ICU stay durations and ER stay duration showed significant differences, particularly in relation to injuries in the lumbar and thoracic regions (*p* < 0.05).

**Conclusion:**

According to the results of the present study, trauma is more severe, and cervical injuries are more common in young people, which is a critical finding that underscores the need for targeted interventions to mitigate the severity of trauma in this age group. Additionally, the majority of cervical injuries occurred in young people, which is a particularly concerning finding given the potential for long-term disability and impact on quality of life. Our findings suggest that strategies to reduce cervical injuries, such as speed control, seat belt use, and phone-free driving, are crucial interventions for mitigating the severity of trauma and promoting patient outcomes in young people.

## Introduction

Trauma refers to any impact, injury, shock, wound, or event that affects the human body due to external causes, as opposed to internal factors like diseases.^1,2^ Such incidents may result in severe complications, disability, and financial and social burdens.^3^ Spinal cord injury (SCI) is a serious medical condition that often results in severe complications and permanent disability. This kind of injury occurs when nerve axons that pass through the spinal cord are disrupted, leading to loss of motor and sensory functions beneath the injured area. Injury is usually the result of major trauma, and the initial injury is often irreversible.^4^ Injuries to the spinal cord are classified as either traumatic [traumatic SCI (TSCI)] or nontraumatic [nontraumatic SCI (NTSCI)], and trauma is the most common cause of SCI globally.^5^ Conti et al.’s study provided insights into the incidence and mortality of SCI in Italy’s Piedmont Region from 2008 to 2020 through a retrospective population-based cohort study. The study found that Italy’s SCI incidence rate is lower than other European countries, with higher rates among males than females, and older adults compared to younger adults. The study also revealed higher mortality rates following SCI in Italy than in other European countries, highlighting an adverse impact on SCI survivors’ and their families’ quality of life.^6^ A study by New et al. highlighted the need to create global maps of NTSCI epidemiology through the establishment of a living data repository. The study analyzed data from 15 countries and discovered that NTSCI is more prevalent in older adults and women than previously thought. The study also identified geographic variations in NTSCI incidence rates, emphasizing the need to address disparities in care and resources between regions with high and low NTSCI incidence rates. The findings of this study underscore the importance of creating a global data repository to facilitate ongoing monitoring and analysis of NTSCI epidemiology and inform policies and strategies to improve NTSCI care and prevention worldwide.^7^

The SCIs are often caused by direct trauma to the spinal cord or pressure by vertebral fractures or masses such as epidural hematoma or abscess.^8^ Normally, the spinal cord may be damaged due to compromised blood flow, inflammatory processes, metabolic disorders, or exposure to toxins.^9^ The classification of SCIs has evolved over the past decades based on a combination of fracture morphology, which is defined by diagnostic imaging in relation to clinical presentation.^10^ SCI is best described into five distinct anatomical regions, each with its own unique anatomy and injury patterns. These regions include the cervical, lower cervical, thoracic, thoracolumbar, and lower lumbar parts of the spine, where most injuries occur.^11^

SCI incidence rates vary globally, with an estimated 250,000 to 500,000 injuries per year worldwide, according to the World Health Organization.^12^ Around 18,000 new cases of SCIs occur annually in the United States, while it is estimated to be around 2,500 in the United Kingdom.^13–15^ The epidemiology of SCI in Western countries has undergone significant changes in recent decades.^6,16^ In this regard, research has indicated that cases of NTSCI exhibit distinct demographic and clinical features compared to TSCI cases. These include a higher median age range of 55–65 years, a less pronounced male-to-female ratio, and etiology related to malignant neoplasms or degenerative conditions.^7^ The aging population has led to a significant rise in the average age of SCI and the occurrence of cervical injuries.^7,17^ In Italy in 2020, the average annual incidence rate was 17.9 cases per million populations.^6^ This has made health policymakers consider modifications to both organizational and clinical rehabilitation approaches.^6,16^ On a global scale, an estimated 9 million SCI cases were reported in 2019, marking a 52.7% increase since 1990. The age-adjusted incidence rate for SCIs in 2019 was 11.5 per 100,000 population, with no significant changes observed in the incidence rate between 1990 and 2019 for both males and females.^17^

According to a single-center study conducted in South Korea, the main cause of TSCI is blunt trauma, which mostly occurs as a result of motor vehicle accidents (48%), falls (21%), and sports injuries (14.6%).^18^ As per recommendation from the World Federation of Neurosurgical Societies Spine Committee recommendations on epidemiology, prevention, and early management of cervical spine injuries, nevertheless, it should be noted that most SCIs (60%) occur in healthy young men aged 15–35 years, and cervical injuries are the most common.^19^ Furthermore, 80% of patients with SCI have multisystem injuries, including other bone fractures (29.3%) and traumatic brain injury (11.5%).^20^ In Scotland, falls are the most common precipitator of SCI, followed by road traffic accidents.^5^ The most common cause of SCI in Western and Central Europe is falls; a common phenomenon in the elderly population.^21^

In the United States, the most common cause of SCI was motor vehicle collisions (MVCs), which accounted for 38% of new SCIs each year, followed by falls (30%), violence (13%), sports injuries (9%), and medical and surgical problems (5%).^22^

The prognosis for patients with SCI is very poor, and there is no definitive treatment for the recovery of SCIs. Less than 1% of patients recover before discharge from the hospital.^23^ The level of disability is directly related to the level of injury. SCI patients suffer significantly higher mortality in the first year after injury, and those who survive still have reduced life expectancy, and merely 12% continue their careers.^24^

SCIs are one of the main causes of disability, which especially affects healthy young people with important socio-economic consequences and causes lifelong care and rehabilitation costs. To gain comprehensive insights into spinal fractures resulting from blunt trauma incidents in Iran, a study was conducted using data collected from 2018 to 2022. This study aimed to ensure a diverse age group representation, thus providing valuable information on the prevalence and characteristics of these injuries across different age groups in the country. Besides determining the frequency of these fractures, the research also explored the injury mechanisms involved as a secondary objective. Upon concluding the analysis, the researchers plan to propose recommendations to help alleviate the issue based on their findings, ultimately contributing to improved prevention and management strategies for spinal fractures resulting from such incidents.

## Methods

This retrospective cohort study was performed on data collected between 2018 and 2022 based on the Trauma Registry data from 14 Trauma Centers, which together constitute the National Trauma Registry of Iran (NTRI). We used this database to identify blunt trauma patients with spinal fractures. The database contains information including demographic information (age, gender, etc.), injury information (cause of trauma, type of trauma, etc.), comprehensive clinical information, diagnostic codes (International Classification of Diseases-Ten Version, ICD-10-CM), severity of injury, and patients’ outcomes. In this study, as the researchers only had access to anonymous data that did not permit the identification of individuals, the Research Ethics Committee of Isfahan University of Medical Sciences approved the utilization of such data without requiring patient consent (Approval No.: IR.MUI.MED.REC.1401.043).

All individuals admitted to the 14 trauma centers from 2018 to 2022, with TSCI as per ICD-10-CM codes S12.0 to S12.2, S12.7, S22.0, S22.1, and S32.0, were eligible for inclusion.^25^ A total of 1,167 patients with blunt trauma in at least one of the cervical, thoracic, or lumbar vertebrae were included in the study by the census sampling method. The exclusion criteria were any blunt fracture other than the cervical and thoracolumbar and brain death patients. Injuries to the sacrum were not considered because the sacrum is a fixed vertebra and is normally considered part of the pelvis. Also, to predict the outcome and likelihood of mortality, the injury severity score (ISS) was used.

The ISS is an anatomical scoring system that provides an overall score for patients with multiple injuries. Every injury is given a score based on the abbreviated injury scale and is assigned to one of the six body regions. The ISS score is calculated by squaring the scores of the three most severely injured body regions and adding them together. The ISS score goes from 0 to 75.^26^

Statistical Package for Social Sciences was used for the statistical analysis (Version 22, IBM Corp., Armonk, NY, USA). In terms of descriptive statistics, means and standard deviations were used for continuous variables and percentages were used for categorical variables. The chi-square test was used to assess the relationship between the variables and to explore the risk factors. A p-value of <0.05 was defined as statistically significant.

## Results

**Figure 1.**
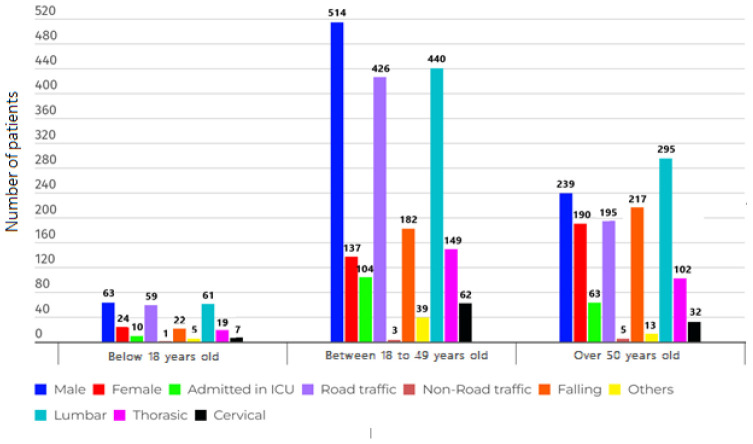
Demographic chart of the distribution of gender, hospitalization, type of accidents, and level of involvement of the vertebral column, categorized by age groups.

From 2018, there were 25,986 traumatic cases registered on the NTRI website. Out of these cases, 1,167 cases (4.5%) were related to blunt traumas and spinal fractures. Table 1 and Figure 1 display the demographic and clinical characteristics of patients categorized by age group. The analysis by gender, severity of injury, and cause of trauma depicted a significant difference among various age groups (*p* < 0.05).

Statistically significant correlation between each age group and the variable cross-named in each row at a significance level of *p* < 0.05.

SD, standard deviation; ICU, intensive care unit, N, number; ER, emergency room: ISS, Injury Severity Score.

Other: cutting injuries, non-cutting injuries, drowning, direct burn, indirect burn, suffocation, poisoning, electrocution, firearms, exposure to blast waves, animal bites, unknown factors, and so on.

**Figure 2.**
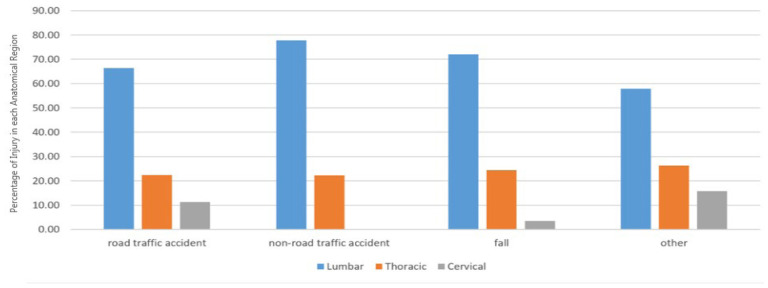
Injury mechanism of traumatic spinal cord injury patients in each anatomical region.

Figure 2 shows the injury mechanism in each anatomical region of the spine. A significant difference was observed between the mechanism of injury in each anatomical region of the spine (*p* < 0.05). Specifically, the lumbar region was more affected by various injury mechanisms compared to other regions.

**Figure 3.**
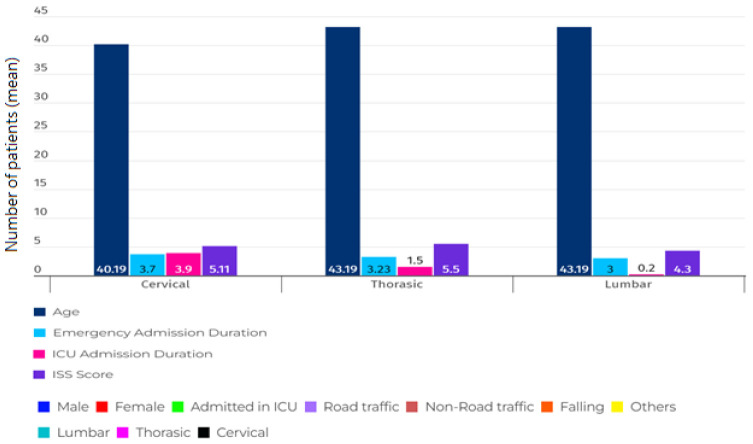
Demographic chart of average age, duration of hospitalization, and severity of injury based on ISS, categorized by the location of vertebral injury.

**Figure 4.**
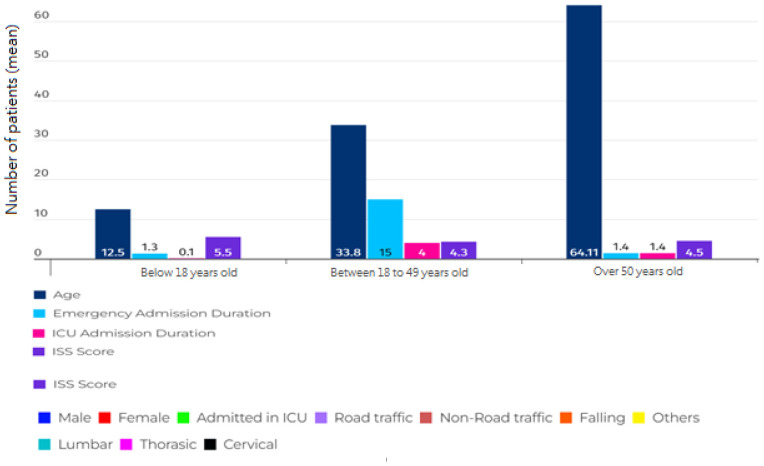
Demographic chart of average age, duration of hospitalization, and severity of injury based on ISS, categorized by age groups.

Table 2 and Figure 3 display the demographic and clinical characteristics of patients categorized by the anatomical region of the spine. We found a direct relationship between the amount of time people stayed in the Emergency Room (ER) and Intensive Care Unit (ICU) and the injury severity in TSCI patients (*p* < 0.05). Figure 4 also shows the demographic chart of average age, duration of hospitalization, and severity of injury based on the ISS score, categorized by age groups.

## Discussion

After analyzing a sample consisting of 1,167 patients with blunt trauma and spinal fracture, according to Table 1, the average age was found to be 43.9 ± 5.5 years, with 816 (69.9%) of them being male. The majority of patients, 796 (68.2%), had lumbar spinal fractures. Road Traffic Collisions (RTCs) with 680 (58.3%) cases and falls with 421 (36.1 %) cases were the most frequent causes of SCI injuries, respectively. The severity of injury mean was equal to 4.4 ± 3.5. In 2021, Iran’s population, as reported by the World Bank, was approximately 83.85 million. The population had a gender distribution with males constituting 47.7% and females making up 52.3%. The United Nations provided age distribution data for 2020, highlighting that the majority were under 65 years old. Among these age groups, the 25–54-year demographic contributed 46.59% to the population, while the 10–24 years age group accounted for 15.74% of the total population. The injury severity score was higher in younger patients (under 18 years old). RTCs were more common in the 19–49 age group, while non-road traffic accidents were more frequent in the age group above 50 years. Men outnumbered women in all age groups. Also, according to the results of Table 1 and Figure 1, falls were reported as the most common cause of injury in men over 50 years old. A statistically significant difference was observed between the causes of injury in each anatomical region of the spine, as shown in Figure 2. The majority of injuries occurred in the lumbar area, followed by the chest area. According to Table 2, there was a statistically significant difference observed between the length of stay in ER, hospitalization and length of stay in the ICU, and the severity of injury by the anatomical region of the spine, the severity of injury being higher in the cervical region. Finally, gender, the severity of injury, and the cause of injury showed a statistically significant difference among different age groups.

Although other studies have been conducted on patients with spinal fractures, few studies have followed the objectives of this study. In 2023, Rayatdoost et al.^27^ conducted a study on patients with SCIs in Jahrom, Iran. The participants had an average age of 40.89 ± 15.85 years, and 78.7% of them were male, and the main causes of injury were reported to be RTCs (58.70%) and falls (33.30%). These findings were consistent with the results of the present study. In 2020, Andalib et al.^28^ evaluated 1,014 patients with TSCI in Isfahan (Iran) and reported that the most common mechanism of trauma was RTCs (83.4%) followed by falls (12.7%), and the lumbar area had the highest incidence of TSCI (38.3%) followed by thoracic spine fractures (27.4%). These results aligned with the outcomes obtained in the current investigation. According to the high agreement between the results of our study and the findings of the two studies of Rayatdoost and Andalib that were conducted in Iran, the similar road accident rates among young people in various parts of Iran could be attributed to a range of factors such as infrastructure, road conditions, driving culture, enforcement of traffic laws, and access to education and resources regarding safe driving practices. Furthermore, driving behaviors and risk-taking tendencies among young people may also be influenced by societal and economic factors in different regions.^29^ In general, a combination of these factors contributes to relatively uniform accident rates among young people in different parts of Iran, despite potential differences in geographic and cultural characteristics. Also, the similar fall rate among individuals over 50 years of age in various parts of Iran could be attributed to several factors such as demographic similarities, lifestyle factors, healthcare access, environmental factors, and public health initiatives.

In another study, Liu et al.^30^ in China presented findings indicating that the average age of individuals with TSCI was 46.3 ± 15.5 years, with a male-to-female ratio of 1:4.73. The main cause of such injuries was falls, accounting for 30.8% of cases. Although the age group was similar in our studies, the cause of injury was different.

In 2018, in Kuwait, Alhadhoud et al.^31^ checked 564 patients who had 788 traumatic injuries. The average age of the patients was recorded as 37.10 years, with males accounting for 79.2% of the cases. The most frequent cause of injury was road accidents with 54.5%, and the lumbar spine was the most common fracture site with 47.5%. Notably, the study identified sex, nationality, anatomical site of fracture, and injury mechanism as significant risk factors. The findings of this study were more similar to the results obtained in the present study.

In the Swedish Fracture Register, 27,169 fractures were reviewed by Bergh et al.^32^ The average age at the time of fracture was 57.9 years (ranging from 16 to 105 years), and the majority of fractures (64.5%) occurred in women. The results of this study were also not consistent with those of the present study. This disparity could potentially be attributed to not only variations in trauma patterns across distinct nations but also to disparities in population demographics. Notably, the higher age of trauma and fractures observed in Bergh’s study may indicate a greater number of incidents occurring among older individuals.

In the other study conducted by Utheim et al.^33^ a total of 2,153 patients with traumatic cervical fractures were reviewed over a span of 5 years in Norway. The average age of the patients was 62 years, with 68% being male. Among the patients, 53% had multiple traumas, while 12% had cervical spinal injuries. Most of the injuries were caused by falling (57%), but this finding was different from what we found in our study. The differences in results can be due to differences in the age groups involved with trauma in the two studies. That is to say, the patients were younger in the current study, so RTCs are the most common mechanism of trauma, but in Utheim’s study, which includes the elderly, falls were the most common mechanism of trauma.^33^

In the year 2018, Passias et al.^34^ reviewed 488,262 patients by using the National Inpatient Sample database for the United States. The mean average age of the patients was 55.96 years and males constituted 60% of the studied patient sample. MVCs and falls were independently associated with cervical spine injury. It was found that MVCs accounted for 29.3% of cases. Moreover, the most prevalent fracture type, observed in 32% of instances, was related to the C2 vertebra. Although the age groups in the two studies were different, the cause of trauma in both studies was MVC.

Consistent with previous research,^31–33,35–39^ our findings indicate a higher incidence of trauma in men compared to women. Furthermore, the finding of this study showed that the most prevalent mechanism of injury in Iran is road accidents, highlighting the need for increased attention to traffic laws in the country. The authors of the above-cited articles suggest implementing measures to increase road safety, such as using cell phone blocking technology, satellite technology, self-driving or connected and autonomous vehicles, Internet of Things, artificial intelligence, and 5G.^38^ They also propose enforcing traffic laws, such as effective punishment, improved police enforcement (with enhanced demerit points for young drivers and drug testing, etc.), educating the public about road safety hazards, and improving young driver culture and attitudes, raising the level of traffic standards, and providing secure transportation facilities to prevent and manage TSCI.^39^ These actions are crucial for managing the growing number of individuals with TSCI as young adults are considered a critical population due to their role in society as the backbone of society, the reserve force, the active force, and the national treasure of society.

### Limitations

Our study has a few limitations. First, due to some incomplete information, the accuracy and reliability of the study’s findings could have been affected as missing or inaccurate data could lead to errors in analysis and interpretation of results. Second, although this study included a relatively large number of patients, its sample size could still be considered relatively small, which could limit its generalizability and applicability to other populations. Third, the accuracy and completeness of the data in the registry may vary depending on the reporting practices of individual hospitals. Some hospitals may have more complete data than others. Finally, it is essential to note that this study focused solely on blunt trauma and spinal fractures as causes of spinal injuries; however, some injuries could also result from other mechanisms such as tumors or infections, which were not addressed in this study. Therefore, future research should aim at addressing these limitations and expanding its scope by including a wider range of spinal injuries and populations in its study design.

## Conclusion

According to the results of the present study, trauma is more severe, and cervical injuries are more common in young people, which is a critical finding that underscores the need for targeted interventions to mitigate the severity of trauma in this age group. Additionally, the majority of cervical injuries occurred in young people, which is a particularly concerning finding given the potential for long-term disability and impact on quality of life. The safety of young drivers, particularly young men, has always been a concern due to their nonobservance of basic safety principles and human errors caused by cultural and gender-related driving behaviors. These factors can lead to mortality or morbidity in themselves or others. Effective management of this issue requires planning for correction.

Also, among individuals aged 50 and above, falls were identified as the primary cause of spinal injuries. To decrease the prevalence of falls, prevention is more effective than medical management. Modifying one’s lifestyle and creating a safe living environment can help prevent falls. In addition to treating fall injuries, fall management should focus on the causative factors. Taken together, we found that the epidemiological features of TSCI vary among different societies due to different causes.

## Table of Abbreviations

**Table:** 

**Abbreviations**	**Term**
SCI	Spinal cord injury
TSCI	Traumatic SCI
NTSCI	Nontraumatic SCI
WHO	World Health Organization
NTRI	National Trauma Registry of Iran
ICD-10-CM	International Classification of Diseases-Ten Version
ISS	Injury severity score
AIS	Abbreviated Injury Scale
SPSS	Statistical Package for Social Sciences
SD	Standard deviation
ICU	Intensive care unit
N	Number
ER	Emergency room
CAV	Connected and automated vehicles
IoT	Internet of Things
AI	Artificial intelligence
TBI	Traumatic brain injury
RTA	Road traffic accident
RTC	Road traffic collision

## Conflict of Interest Statement

The authors declare that they have no conflict of interest.

## Acknowledgments

We thank the National Trauma Registry of Iran, affiliated with Sina Trauma and Surgery Research Center, and Isfahan Trauma Data Registration Center. We also wish to thank the Vice Chancellery for Research at Isfahan University of Medical Sciences for providing support for this study with the research project number: 340144.

## Authors’ Contributions

MNI, PS, and RA contributed to the conception and design of the work. NF and SN helped with data collection. Data interpretation, drafting, and critical revision of the paper were conducted mainly by SN, NF and MNI. All authors read and approved the final version of the article.

## Data Availability

The data that support the findings of this study are available on reasonable request from the corresponding author. The data are not publicly available due to their containing information that could compromise the privacy of research participants.

## Figures and Tables

**Table 1.** Demographic and clinical characteristics of patients with traumatic spinal cord injury by age group.

**Variables**		**Total number**	**<18 years** **(*n* = 87)**	**18–49 years** **(*n* = 651)**	**≥ 50 years** **(*n* = 429)**	***p*-value**
Age (in Years) (Mean ± SD)		43.9 ± 5.5	12.5 ± 9.3	33.8 ± 5.3	64.11 ± 9.4	0.0001
Gender, N (%)	Male	816 (69.9)	63 (7.7)	514 (63)	239 (29.3)	0.0001
	Female	351 (30.1)	24 (6.8)	137 (39)	190 (54.2)	
Length of stay in the ER (days) (Mean ± SD)		1.11 ± 7.7	1.3 ± 4.5	15 ± 2.2	1.4 ± 3.3	0.626
Number of patients admitted to the ICU, N (%)		177 (15.9)	10 (5.6)	104 (58.8)	63 (35.6)	0.500
Length of stay in ICU (days) (Mean ± SD)		4 ± 1.4	0.1 ± 4.3	4 ± 1.5	1.4 ± 1.7	0.405
ISS (Mean ± SD)		4.4 ± 3.5	5.5 ± 1.8	4.3 ± 2.4	4.7 ± 5	0.001
	Road traffic accidents	680 (58.3)	59 (8.7)	426 (62.6)	195 (28.7)	
Cause of trauma, N (%)	Non-road traffic accidents	9 (0.8)	1 (11.1)	3 (33.3)	5 (55.6)	0.0001
	Fall	421 (36.1)	22 (5.3)	182 (43.2)	217 (51.5)	
	Other	47 (4.8)	5 (5.7)	39 (68.4)	13 (22.8)	
	Lumbar	796 (68.2)	61 (7.6)	440 (55.3)	295 (37.1)	
Type of fracture, N (%)	Thoracic	270 (23.1)	19 (7)	149 (55.2)	102 (37.8)	0.814
	Cervical	101 (8.7)	7 (6.9)	62 (61.4)	32 (31.7)	

**Table 2.** Demographic and clinical characteristics of patients by the anatomical region of the spine.

**Variables**		**Lumbar** **(*n* = 796)**	**Thoracic** **(*n* = 270)**	**Cervical** **(*n* = 101)**	***p*-value**
Age (Mean ± SD)		43.19 ± 8.6	43.1 9 ± 7.1	40.19 ± 9.5	0.383
Gender, N (%)	Male	546 (66.9)	192 (23.5)	78 (9.6)	0.181
	Female	250 (71.2)	78 (22.2)	23 (6.6)	
Length of stay in the ER (days) (Mean ± SD)		3 ± 1.5	3.23 ± 1.1	3.7 ± 2.9	0.019
Number of patients admitted to the ICU, N (%)		101 (69)	51 (28.8)	25 (14.1)	0.0001
Length of stay in ICU (days) (Mean ± SD)		0.2 ± 6.8	1.5 ± 6.2	3.9 ± 2.3	0.0001
ISS (Mean ± SD)		4.3 ± 3.2	5.5 ± 5.3	5.11 ± 9.4	0.0001
